# Artificial intelligence powered advancements in upper extremity joint MRI: A review

**DOI:** 10.1016/j.heliyon.2024.e28731

**Published:** 2024-03-25

**Authors:** Wei Chen, Lincoln Jian Rong Lim, Rebecca Qian Ru Lim, Zhe Yi, Jiaxing Huang, Jia He, Ge Yang, Bo Liu

**Affiliations:** a Department of Hand Surgery, Beijing Jishuitan Hospital, Capital Medical University, Beijing, China; b Department of Medical Imaging, Western Health, Footscray Hospital, Victoria, Australia; c Department of Surgery, The University of Melbourne, Victoria, Australia; d Department of Hand & Reconstructive Microsurgery, Singapore General Hospital, Singapore; e Institute of Automation, Chinese Academy of Sciences, Beijing, China; f School of Artificial Intelligence, University of Chinese Academy of Sciences, Beijing, China

**Keywords:** Artificial intelligence, Deep learning, Convolution neural network, Upper extremity, Magnetic resonance imaging

## Abstract

Magnetic resonance imaging (MRI) is an indispensable medical imaging examination technique in musculoskeletal medicine. Modern MRI techniques achieve superior high-quality multiplanar imaging of soft tissue and skeletal pathologies without the harmful effects of ionizing radiation. Some current limitations of MRI include long acquisition times, artifacts, and noise. In addition, it is often challenging to distinguish abutting or closely applied soft tissue structures with similar signal characteristics. In the past decade, Artificial Intelligence (AI) has been widely employed in musculoskeletal MRI to help reduce the image acquisition time and improve image quality. Apart from being able to reduce medical costs, AI can assist clinicians in diagnosing diseases more accurately. This will effectively help formulate appropriate treatment plans and ultimately improve patient care. This review article intends to summarize AI's current research and application in musculoskeletal MRI, particularly the advancement of DL in identifying the structure and lesions of upper extremity joints in MRI images.

## 1 Introduction

Magnetic resonance imaging (MRI) is an indispensable imaging modality in musculoskeletal medicine. Since the first MRI image of humans was obtained in 1977, MRI has been widely used in assessing skeletal and soft tissue structures because of its non-ionizing radiation [1]. MRI imaging techniques have been constantly changed and optimized in the following decades. The field strength has gradually evolved from the original 0.25-T to the current mainstream 1.5-T and 3.0-T, as well as the newly emerged 7.0-T in recent years [2]. MRI sequences gradually evolve from conventional T2WI and T1WI to fast spin-echo (FSE), proton density-weighted imaging (PDWI), fat-suppressed (FS), etc. Recently, the emergence of the 3D MRI reconstruction technique provides more application prospects for the post-processing of musculoskeletal MRI scanning [3].

Artificial Intelligence (AI), once in the realm of science fiction, has gained traction in every aspect of modern life. As a branch of AI, machine learning (ML) is usually used to solve two problems: classification and regression. Segmentation describes an image's regions of interest (ROI) and can be regarded as pixel-level classification. Image reconstruction, a sort of pixel-level regression, outputs a continuous pixel array to reconstruct clinical images from MRI raw data in K-space. Regression and classification can be achieved through both supervised learning and unsupervised learning. As a sub-domain of ML, Deep Learning (DL) simulates human cognitive reasoning via functional units of artificial neurons interconnected through complex neural networks. The process of learning to perform tasks is interpreted as model training, and the ultimate goal of training is to minimize the error between the predicted results of the model and the ground truth. As a feed-forward neural network, the convolution neural network (CNN) considers the spatial position of pixels and manually sets the degree of interest (weight) to achieve weight sharing based on traditional neural networks. CNN has been successfully applied in various fields, especially machine vision (including image detection, location, classification, and segmentation). With more data generation and dataset availability on the horizon, ML and DL have greatly benefited and progressed medical image processing and analysis.

Conventional MRI image acquisition acceleration techniques include parallel imaging (PI), three-dimensional (3D) isotropic turbo spin-echo (TSE) imaging, simultaneous multi-slice (SMS) acquisition, and compressed sensing (CS) undersampling [[3], [4], [5], [6]]. However, these acceleration techniques have shortcomings, such as low signal-to-noise ratio (SNR) and artifacts caused by acceleration [5,7,8]. While motion-corrected periodically rotated overlapping parallel lines with enhanced reconstruction (PROPELLER, a kind of radial k-space acquisition technique) has been widely employed to correct spatial variations and reduce motion artifacts, it is usually accompanied by an increased scan acquisition time [9]. In 2013, 2D CNN implemented the segmentation of tibial cartilage in low-field knee MRI scans for the first time [10]. In the following period, more DL architectures have been developed and optimized to obtain higher-quality MRI images to achieve better diagnostic performance [11,12]. However, these studies are more focused on the knee joint. In musculoskeletal MRI, DL applications include end-to-end image networks that allow datasets with higher acceleration coefficients to be reconstructed into clinically available images through advanced denoising, aliasing, section leakage correction, coil corrections, and super-resolution interpolation [11,13]. In recent years, some DL reconstruction (DLR) products for MRI have been developed and put into musculoskeletal clinical applications, such as AiCE, developed by Canon Medical Systems Corporation, and AIR™ Recon DL, created by GE Healthcare [[14], [15], [16], [17], [18]]. Studies have shown that a combination of DLR techniques and conventional MRI reconstruction techniques have great potential in 1) eliminating image artifacts caused by the conventional accelerated acquisition techniques, reducing scanning time without SNR loss; 2) maintaining an accurate representation of anatomy, diagnostic confidence, edge sharpness, and detection of upper extremity pathologies; 3) increasing patient comfort and patient throughput, decrease medical cost and energy consumption [8,14,15,[17], [18], [19], [20], [21], [22]]. Table 1 lists the detailed comparison of conventional MRI acceleration or super-resolution and DLR techniques.

**Table 1:** Comparsion of conventional MRI acceleration or super-resolution technique and DLR technique.

Conventional technique	Interpretation	Disadvantages	Author	Year	Joint	MRI sequence	DLR technique	Scan time	DLR advantages summary
PI	Achieved by sampling every second or third k-space line during image acquisition, with the degree of acceleration proportional to the reduction in scanning time.	Increased noise and artifacts	Hahn et al. [16]	2021	Shoulder	2D FSE	DLR pipeline (based on CNN) + PI	67% reduction compared to standard 2D FSE sequences	1. Eliminate image artifacts caused by the conventional accelerated acquisition techniques and reduce scanning time without SNR loss. 2. Maintain an accurate representation of anatomy, diagnostic confidence, edge sharpness, and detection of pathologies. 3. Increase patient comfort and patient throughput and decrease medical cost and energy consumption.
			Liu et al. [8]	2023	Shoulder	T2WI-FS, PD-FS	Modified U-net CNN + PI	50% reduction compared to PI alone	
			Herrmann et al. [20]	2023	Wrist	TSE	DLR + PI	60% reduction compared to standard fully sampled TSE sequences	
			Herrmann et al. [21]	2023	Elbow	TSE	DLR + PI	35% reduction compared to standard TSE sequences	
CS	Based on disproportionate undersampling of less contributory data in sparse regions with little if any unique imaging information.	Increased noise and artifacts	Obama et al. [15]	2022	Shoulder	PD-FSE	AiCE + PI + CS	52% reduction compared to PI alone	
			Shiraishi et al. [14]	2023	Shoulder	T1WI, T2WI, T2WI-FS	AiCE (DLR + CS + wavelet denoising)	/	
			Feuerriegel et al. [22]	2023	Shoulder	TSE	DL-based ISTA + CS	no difference compared to CS	
PROPELLER	A kind of radial k-space acquisition technique to correct spatial variations and reduce motion artifacts.	Increased scan acquisition time	Kaniewska et al. [17]	2022	Shoulder	T1WI-FS, T2WI-FS, PD-FS	AIR Recon DL pipeline (based on CNN) + PROPELLER	62% reduction compared to PROPELLER alone	
			Hahn et al. [18]	2023	Shoulder	2D FSE	AIR Recon DL pipeline (based on CNN) + PROPELLER + PI	45% reduction compared to PROPELLER alone	

Abbreviations: DLR, deep learning reconstruction; PI, parallel imaging; CS, compressed sensing; PROPELLER, periodically rotated overlapping parallel lines with enhanced reconstruction; T1WI, T1-weighted imaging; T2WI, T2-weighted imaging; TSE, turbo spin echo; FSE, fast spin echo; PD, proton density; FS, fat-suppressed; AiCE, Advanced intelligent Clear-IQ Engine; ISTA, iterative shrinkage threshold algorithm; SNR, signal-to-noise ratio.

AI has progressed tremendously since its inception and has been applied in musculoskeletal imaging in recent years. This article aims to provide an extensive review and summary of the current progress of AI-assisted identification and diagnosis of upper extremity joint structures and diseases in MRI images for clinicians, surgeons, radiologists, and digital health experts.

## 2 Methods

The primary objective of this study was to provide a comprehensive overview of AI's current research and application in musculoskeletal MRI, particularly the advancement of DL in identifying the structure and lesions of upper extremity joints in MRI images. This article also aims to help clinicians, surgeons, radiologists, and digital health professionals navigate and deepen their understanding of this exciting medical imaging progress. To achieve this, we scrutinized literature from various sources, mainly including peer-reviewed journal articles, conference papers, etc. [23,24].

In exploring studies related to AI in upper extremity joint MRI, comprehensive searches were performed on Web of Science, PubMed, and Scopus using the following items: (‘Artificial intelligence’ OR ‘AI’ OR ‘deep learning’ OR ‘machine learning’ OR ‘convolutional neural network’ OR ‘CNN’) AND (‘shoulder’ OR ‘wrist’ OR ‘elbow’) AND (‘magnetic resonance imaging’ OR ‘MRI’). We conducted a preliminary screening of the retrieved literature and included the closely relevant studies to construct this narrative review.

The overall outline of this review is as follows. Sections 3, 4 present a comprehensive overview of advancements in AI technologies in segmenting musculoskeletal anatomical structures and assisting in diagnosing diseases on shoulder and wrist MRI. Section 5 clarifies the limitations and challenges of implementing AI in MRI, including the potential for AI bias, the need for large datasets for training, ethical considerations, and data privacy concerns. Section 6 discusses emerging AI technologies and potential future research directions that could impact musculoskeletal MRI imaging and concludes and emphasizes the medical benefits of AI technologies.

## 3 Shoulder

Shoulder pain, which usually results from lesions of the rotator cuff (RC) and other soft tissue structures, is debilitating and causes significant inconvenience to daily life, particularly if it affects the dominant limb. While ultrasound is a convenient and harmless form of imaging assessment, the MRI scan is still the preferred and gold standard imaging modality to detect shoulder lesions. MRI sequences can vary from institution, but the most used standard scanning sequences for shoulder imaging include 2D FSE, T1WI, PDWI, or three-plane T2WI. Due to lengthy scanning time and patient discomfort, there is a higher propensity for motion and respiratory artifacts [25]. In addition, there is also increased image noise (for example, Rician noise) and limitations on surface coils, which often reduce the acquired image quality [26]. DL-based shoulder MRI acceleration, classification, and reconstruction can shorten the image acquisition time and cost. Other benefits include higher-quality images and quick identification of pathology, which would ultimately guide clinicians to formulate the appropriate treatment plans swiftly.

### 3.1 DL for RC structure identification and RC tears diagnosis

20–50% of people over 60 suffer from RC tears (RCT). Fatty infiltration (FI) and RC muscle atrophy have also been shown to greatly impact RC surgical repair outcomes [27,28]. Occupation ratio (OR), which is the ratio between the surface of the cross-section of the muscle belly and that of the fossa, usually determines supraspinatus muscle atrophy [29]. The measurement of OR is usually complex and labor-consuming, especially for RC muscles with irregular margins [30]. In addition, the Goutallier scoring system most commonly used to evaluate FI is subjective and non-quantitative [31,32].

MRI examinations are often used to assess the crucial features of RCT to help guide Orthopedic management [33]. Accurate segmentation and reconstruction of RC muscles, especially the frequently involved supraspinatus, is quintessential for surgical planning and postoperative outcomes. On conventional MRI techniques, it is often challenging to segment and assess the complex structures with high contrast near the supraspinatus and their boundaries [34]. CNN structure can help achieve high-quality segmentation of complex structures by pre-training the model to follow the global anatomical characteristics of shape and position, which is highly useful for shoulder MRI image segmentation.

#### 3.1.1 2D and 3D CNN model

**Fig. 1:**
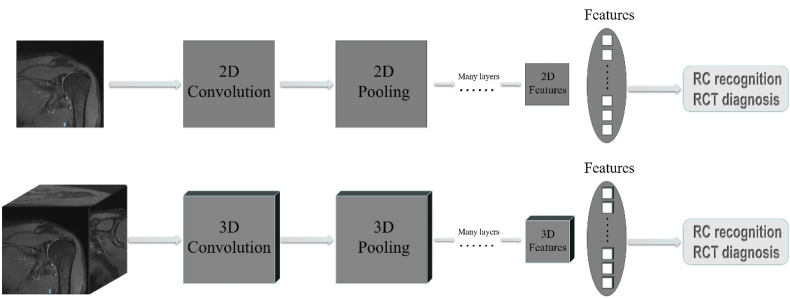
A schematic diagram of the 2D and 3D CNN models. For 2D CNN, a single image slice was the input, while 2D convolution layers were utilized to extract image features and then fed into a classifier, from which the output realized RC recognition or RCT diagnosis. For the 3D CNN model, several image slices from different views (transverse, coronal, and sagittal) or the reconstructed 3D blocks were the input, and 3D convolution layers were utilized to extract image features and then fed into a classifier, from which the output realized RC recognition or RCT diagnosis.

Two CNN models, namely 2D and 3D CNN, have been employed for image segmentation. 2D CNN uses 2D convolution and pooling layers, which take a single slice in a single MRI view as input. 3D CNN employs 3D convolution and pooling layers, which takes cuboids across several MRI slices of three MRI views as input. 3D CNN can obtain more information from multiple views and simulate the diagnostic process used by radiologists [35]. Fig. 1 shows a schematic diagram of the two CNN models.

#### 3.1.2 2D CNN for classification and quantification

This part will discuss the current applications of 2D CNN in MSK MRI imaging. In a 2D residual U-Net architecture, images were decomposed from low-level to high-level step by step to extract essential information from the images [36]. To avoid model overfitting and remove any bias in evaluations, the datasets included 157 subjects and were divided into three groups: training dataset (70%), validation dataset (10%), and test dataset (20%). Although 2D residual U-Net automatically realized individual RC muscle segmentation and showed better performance in segmenting muscles with well-defined boundaries, this study included some low-quality MRI scans, which may adversely affect the model's performance. In addition, incorporating more samples from multiple institutions as external test datasets will improve the generalizability of 2D residual U-Net. Differently, Medina et al. [37] utilized Inception v3 architecture to select oblique sagittal MRI images (Y-shaped) (accuracy >0.98) before achieving automatic segmentation of RC muscles using a modified U-Net architecture (DSC >0.93). However, the proposed two-stage method could vary in a cohort with a higher prevalence of severe RC muscle atrophy (low OR) due to the bias of datasets.

Esfandiari et al. [38] designed a 2D CNN based on trial and error and achieved higher accuracy than the pre-trained MobileNetv2 and SqueezeNet on RCT diagnosis. In this architecture, the number of convolutional layers and their filters was evaluated by trial and error, and the average accuracy, sensitivity, and specificity reached 92.67%, 91.75%, and 92.22%, respectively. A 2D CNN framework combined with a three-network design can also accurately detect supraspinatus tears automatically [39]. First, the Slice Selection Network based on ResNet was used to select image range where supraspinatus is visible; second, the Segmentation Network based on U-Net realized 2D slice-wise supraspinatus segmentation; third, the Classification Network was used to recognize if tear presents. The three-network method included a total of 200 patients' MRI images (80% for training and optimization, 20% for test) containing a full-thickness tear, partial-thickness tear, or intact supraspinatus tendon, and the sensitivity, specificity, and DSC were 0.85, 0.85, 0.814, respectively. However, the lack of the gold standard of arthroscopy and the verification of external datasets is a great challenge to the model's generalization ability.

The Extreme Gradient Boosting (XGBoost) algorithm, an ML model, is an ensemble tree-based approach that sequentially builds trees to reduce the errors of the previous trees. In the XGBoost model, it was found that direct signs relating to the tendon signal and morphology were most predictive for subscapular tears on MRI, which demonstrated excellent performance with an accuracy of 0.85 [40]. Furthermore, the addition of physical exam or patient characteristics did not make a significant difference in the predictive ability of this model. Guo et al. [35] further compared the performance between 2D and 3D Xception in diagnosing supraspinatus tears. The results showed that 2D CNN was superior in picking up supraspinatus tears (accuracy 0.87 vs 0.71) and had comparable performance to senior clinicians. It is worth noting that the 2D model discriminates each slice at every view, while the 3D model only discriminates the 3D volume images composed of three views, which contains multiple slices that may be judged as “normal” or “tear” by the 2D model. For a 3D model whose lesions took up only a small part of the whole imaging volume, the high SNR of a 2D model may reach higher qualitative diagnostic performance.

AI is also relevant in the assessment and segmentation of the supraspinatus fossa. The FI and OR can be calculated using the AI method to assist in the diagnosis of RCT. Kim et al. [41] added three fully convolutional layers to the VGG-19 network, which can obtain high-resolution output using skip layers. The proposed fully convolutional network (FCN) realized supraspinatus OR quantification, and the accuracy and DSC in the fossa region and muscle region were 0.9984, 0.9718, and 0.9988, 0.9463, respectively. In another study, the VGG-19-based CNN model was developed to accurately classify the FI to diagnose RCT [42]. However, this study used a binary classification method different from the most commonly used Goutallier classification system. Similar to Kim et al. [41], Ro et al. [43] employed a VGG-19-based FCN model to segment and quantify supraspinatus fossa and muscle. This architecture used 240 shoulder MRI data, of which 90% as training datasets and 10% as validation datasets, and the DSC and accuracy in supraspinatus fossa and supraspinatus muscle were 0.97, 0.9984, and 0.94, 0.9989, respectively. Additionally, an automated region-based Otsu thresholding technique with unsupervised characteristics was developed to quantify fat. Then, RCT can be diagnosed by combining OR and FI parameters. Such a quantified RCT diagnosis mode based on AI overcomes the limitation of being subjective and non-quantitative of the conventional manual Goutallier scoring system. It has the advantages of being more accurate, faster, and labor-saving. These results are helpful for clinicians to make treatment plans, and to evaluate and follow up the postoperative efficacy of patients through the quantitative data of OR and FI on MRI.

2D sequences are often utilized in MRI scanning of the shoulder joint due to its advantages of short acquisition time and fewer motion artifacts. In addition, the 2D CNN model only requires lightweight computation, which is crucial for dealing with complex datasets. However, the measurement on a single 2D sequence has some limitations due to the nonuniform FI throughout the muscle and the low correlation between the resulting Goutallier grade and the real 3D FI measurement [44,45]. While it is easy to diagnose RCT on MRI using 2D CNN reconstructions, it cannot achieve 3D examination or volume quantification of RCTs due to the reliance on a single image but not the entire 3D anatomy. 3D CNN can effectively solve this problem and describe RCT lesions in more volumetric detail.

#### 3.1.3 3D CNN for classification and quantification

The level set segmentation approach used to track the interface is only suitable for images with well-recognized edges, but not for the supraspinatus surrounded by complex structures with high contrast. Therefore, a two-stage active contour segmentation technique using the level set approach that combines internal shape fitting and autocorrection was developed to solve this problem [34]. It can accurately extract the 3D configuration of the supraspinatus and realize in extracting shapes and locating the contours on strong boundaries even for a small-sized supraspinatus (0.995 accuracy and 0.951 DSC). However, it only applies to the data as long as the slice thickness is sufficiently small so that the shapes of the targets in the slices do not rapidly change. Furthermore, to address the challenge posed by the similar signal strength observed in MRI scans of cartilage and tendon, the 3D nnU-Net with secondary labels was performed [33]. Although external validation was exerted and the overall DSC of the reconstructed 3D images was approximately 0.83, the number of patients was relatively small (only 56). Fully automated segmentation of pathological shoulder muscles is challenging due to the considerable variability in muscle shape, size, location, texture, and injury. To segment every RC muscle accurately, Conze et al. [46] expanded the convolutional encoder-decoder network using a VGG-16 encoder pre-trained on ImageNet. They were able to improve the accuracy of the standard U-Net structure (the DSC of 0.824, 0.820, 0.710, and 0.828 for deltoid, infraspinatus, supraspinatus, and subscapularis muscles), achieve automatic segmentation of pathological RC muscles based on learning from healthy to pathological datasets.

It is more intuitive and accurate to realize the classification and quantification of RCT in a 3D MRI view. Voxception-ResNet (VRN) using 3D convolution filters was trained and tested to classify RCT into five classes in a dataset containing 2124 patients' MRI data, outperforming specialized orthopedists in binary accuracy (92.5% vs. 76.4% and 68.2%) [47]. Moreover, a 3D class activation map (CAM) was employed to address the “black box” problem of CNN, visualizing the localization and size information of RCT. In another study, a CNN architecture ensemble (comprising four parallel 3D ResNet50) was used to distinguish between full-thickness tears, partial-thickness tears, and no tears of RC on four MRI views [48]. The model performed best with AUCs of 0.98, 0.99, and 0.95 for full-thickness supraspinatus, infraspinatus, and subscapularis tears, respectively. Due to the different AUC of every RC muscle in different views, this potentially means that the model has relative flexibility in explaining the misidentification of tendon tears caused by protocols, available sequences, incomplete examination, and artifacts. For quantification, a 3D CNN based on U-Net trained in a dataset containing 303 patients’ MRI data was able to perform 3D detection, segmentation, and visualization of RCT lesions, and the DSC, sensitivity, and specificity were 0.943, 0.971, and 0.950, respectively [49]. The model can automatically depict the shape of the tear, maximal tear distance, and volume. Furthermore, it can segment a 2D RCT lesion in multi-planar reformats, enabling clinicians to visualize the lesions in coronal, axial, and sagittal planes.

Different from the Otsu threshold technique for FI quantification and 2D images as input proposed by Ro et al. [43], the two-stage AI model based on 3D U-Net proposed by Riem et al. [50] first segments muscle and bone, and then obtains the FI of every RC muscle. The two-stage AI model realized 3D segmentation and quantification with a DSC of 0.92 for all four RC muscles and their respective FI in clinical MRI images. In addition, the proposed model found the correlation that RC muscle size decreases with age and FI is more predominant in the female group [51]. However, this segmentation method is based only on datasets from two centers and utilizes only sagittal MRI images. Due to the limited field of view, the analysis of OR and intramuscular FI is also consequently limited. In a bid to reduce the time of manual segmentation and slice-by-slice verification, automatic label-specific error checking based on uncertainty has been employed in the 3D nnU-Net, which takes advantage of multiple segmentations generated from both the nnU-Net subnetworks and dropout during inference [52]. The presented model was trained and tested on 76 RCT patients acquired from 19 centers to assess the humerus, scapula, and RC muscles in all MRI views with an average DSC of 0.91. It is hoped that 3D CNN for segmentation, classification, and quantification will be able to widely use automatic 3D RC analysis, eliminate measurement errors caused by 2D CNN, and achieve a more accurate and comprehensive analysis of RC anatomy, thus guiding the optimization of clinical diagnosis and treatment.

### 3.2 DL for other shoulder joint structure identification and disease diagnosis

Besides assessing soft tissue pathology, MRI can also be used to detect the osseous and marrow pathologies of the shoulder joint. However, it is challenging to segment the skeletal composition of the shoulder girdle well due to similarities of MRI signal characteristics, low image SNR, and partial volume effect. To address this issue, an automatic segmentation method combining pulse-coupled neural network (PCNN) and FCN was developed by constructing the block-based AlexNet segmentation model and U-Net-based bone segmentation module [53]. In the proposed architecture, U-Net was used to automatically detect and classify the bone regions in MRI, and AlexNet was used to segment the bone edges in the classified bone regions. An average verification accuracy of 94% was achieved on the humerus and shoulder girdle segmentation on a minimal dataset. Building on this, the algorithm has been integrated into the medical image measurement and analysis platform "3DQI", through which the 3D segmentation effect of shoulder joint bones can be displayed, and it can provide clinical diagnosis guidance to orthopedics [54].

Superior labrum anterior-posterior (SLAP) injury is another common shoulder disease commonly assessed on MRI. While magnetic resonance arthrography (MRA) is considered the gold standard pre-operative assessment for SLAP lesions, the sensitivity and specificity of the scan are not high. Ni et al. [55] developed an MRA-based SLAP-Net model for identifying SLAP injury, which reached an excellent diagnostic performance comparable to senior radiologists. In this model, MRA data from 636 patients were used for training and testing with an accuracy of 0.92 and 0.85, respectively. Other applications of DL have been extrapolated to automated classification of RCT and biceps tendinosis (BT) by Key et al. [56] who created a fixed-size patch-based (PB) model called ViVGG-19 based on the transfer learning model VGG-19. DL has also been applied to RC disorders in obstetrical brachial plexus palsy (OBPP) [46]. Table 2 lists the specific information of studies about the application of DL in shoulder MRI.

**Table 2:** **The detailed information of studies about the application of CNN in shoulder MRI**.

Author	Year	Method	Network architecture	MRI sequence	Imaging view	DSC	Accuracy	Sensitivity	Specificity	Patients/images dataset	Application
Kim et al. [41]	2019	2D FCN	VGG19	T1WI	Y-shaped	0.946 ± 0.047	–	–	–	240	Supraspinatus OR quantification
Conze et al. [46]	2020	3D CNN	VGG-16, U-Net	T1WI-GE	Axial, coronal and sagittal	0.824, 0.820, 0.710, 0.828	–	–	–	12	RC muscles segmentation
Medina et al. [37]	2020	2D CNN	Inception-v3, U-Net	T1WI	Y-shaped	>0.93	>0.96	>0.96	–	258	RC muscles segmentation
Shim et al. [47]	2020	3D CNN	Voxception, ResNet	T1WI-SE, T2WI-SE	Axial, coronal and sagittal	–	0.94 ± 0.02	0.94 ± 0.03	0.90 ± 0.04	2142	RCT degree Classification
Wang et al. [53]	2020	2D PCNN and FCN	U-Net, AlexNet	–	Coronal	0.96 ± 0.02	–	0.94 ± 0.03	0.97 ± 0.02	800	Bone structure segmentation
Mu et al. [54]	2021	2D CNN	U-Net, AlexNet	FS-T1WI-FSE	Coronal	0.91 ± 0.02		0.95	0.95	800	Bone structure segmentation
Ro et al. [43]	2021	2D FCN	VGG19	T2WI	Y-shaped	0.94	–	0.933	0.999	240	Supraspinatus OR and FI quantification
Gwak et al. [33]	2022	2D and 3D CNN	nnU-Net	–	Coronal	0.807, 0.858, 0.978, 0.808	–	–	–	56	Cuff tendon, muscle, bone, and cartilage segmentation
Key et al. [56]	2022	2D CNN	ViVGG19	FS-T2WI	Axial, coronal, and sagittal	–	–	–	–	112	RCT and biceps tendinosis detection
Yao et al. [39]	2022	2D CNN	ResNet, U-Net	FS-T2WI	Coronal	0.814	–	0.85	0.85	200	Supraspinatus tears detection
Lin et al. [48]	2022	3D CNN	ResNet50	T1WI-TSE	Axial, coronal, and sagittal	–	–	–	–	11925	RCT detection and classification
Alipour et al. [36]	2023	2D CNN	Residual U-Net	–	Y-shaped	0.76, 0.83, 0.87, 0.84	–	–	–	157	RC muscles segmentation
Esfandiari et al. [38]	2023	2D CNN	CNN based on trial and error	T1W-SE, T2W-SE	Axial, coronal	0.927	0.911	0.918	0.922	150	RCT detection
Lee et al. [49]	2023	3D FCN	U-Net	FS-PD, PDW-SPAIR	Axial, coronal and sagittal	0.943	0.849	0.971	0.95	303	Posterosuperior RCT detection
Guo et al. [35]	2023	2D and 3D CNN	Xception	T1WI	Oblique coronal	–	0.75	0.913	0.848	829	Supraspinatus tears detection
Hess et al. [52]	2023	3D CNN	nnU-Net	FS-T2WI/PD, STIR	Axial, coronal and sagittal	0.91 ± 0.06	0.81	1	0.94	171	Humerus, scapula, and RC muscles segmentation
Ni et al. [55]	2023	2D CNN	SLAP-Net	MRA, FS T1WI-FSE	Axial, oblique coronal, and Y-shaped	–	0.96	0.804	0.907	636	SLAP detection
Riem et al. [50]	2023	3D CNN	U-Net	T1WI	Y-shaped	0.92 ± 0.14	–	–	–	232	RC muscles and FI quantification
Saavedra et al. [42]	2023	2D CNN	VGG-19, Inception-v3, ResNet-50	T2WI	Y-shaped	–	0.931	1	0.925	606	Supraspinatus FI detection
Cui et al. [57]	2023	2D CNN and Transformer	U-Net, Densnet101, Resnet50, Swin Transformer	FS-T2WI	Coronal	–	0.929	0.918	0.94	431	Supraspinatus tears detection

**Abbreviations:** CNN, convolution neural network; FCN, fully convolutional network; PCNN, pulse coupled neural network; T1W, T1-weighted imaging; T2W, T2-weighted imaging; TSE, turbo spin echo; FSE, fast spin echo; PD, proton density; FS, fat-suppressed; GE, gradient-echo; STIR, short time inversion recovery; SPAIR, spectral attenuated inversion recovery; MRA, magnetic resonance angiography; DSC, Dice similarity coefficient; OR, occupation ratio; RCT, rotator cuff tears; FI, fat infiltration; SLAP, superior labrum anterior-posterior.

## 4 Wrist and elbow

The wrist is a complex anatomical structure capable of performing an intricate range of movements. Due to the anisotropic voxel size of 2D sequences, wrist MRI scans are often time-consuming as the multi-planar images must be acquired separately. As there are various positions that patients would need to adopt for the wrist MRI, the accompanying motion artifacts will lead to poorer image quality and increased scan time and cost. Similar to the PROPELLER technique, TSE (or FSE) also belongs to the spin echo sequence, which is often used in MRI scanning of the wrist and elbow. However, it has the shortcomings of high fat signal, reduced tissue contrast, and blurring effect. Newer 3D SE techniques allow thin-section imaging with isotropic voxels and no interslice gaps [58]. Nevertheless, the unique “Superman” position makes patients uncomfortable, and some patients can't stand this position and finish the examination [59,60]. DLR-based wrist and elbow MRI images can help improve diagnostic image quality and edge clarity [20,21]. It can also significantly reduce acquisition time, thus increasing patient comfort and patient throughput.

### 4.1 DL for wrist structure segmentation

Segmentation, the process of recognition and delineation of structure within the ROI, is crucial for the assessment of tiny complex wrist structures on MRI. Manual labeling is time-consuming, laborious, and vulnerable to high inter- and intra-observer variability [61]. To address this issue, an automatic ML method based on marginal space learning (MSL) and maximum a-posteriori (MAP) labeling was proposed for the first time in 2011 to segment carpal bones in arthritis patients' MRI [62]. Furthermore, a wrist MRI image segmentation toolkit named "WRIST", based on 3D Slicer software, has been developed [63]. The shape, length, area, and volume of wrist bones can also be accurately measured; this enables clinicians to detect dynamic changes, disease evolution, or treatment on MRI imaging. In 2020, allowing for limited training data, CNN utilized a planar architecture and patch-based (PB) training approach (PB–CNN) and significantly outperformed the image-based U-Net network in the wrist cartilage segmentation task (DSC of 0.86 and 0.64, respectively), and showed good consistency with manual segmentation [64]. However, PB-CNN struggled to segment a small number of cartilages away from the medial cross-section ROI due to poorly defined morphological features. Perhaps a 3D CNN architecture may be required to overcome this limitation. Another limitation of PB-CNN would be the heterogeneity of the training datasets, which can affect the segmentation accuracy by mistakenly identifying skin and blood vessels as cartilage on MRI. To improve the network's ability to ignore the image areas outside the wrist joint, Vladimirov et al. [65] integrated attention layers into the skip connections of the U-Net. The results showed that U-Net with attention layers was superior to PB-CNN (DSC of 0.811 vs 0.690) in cartilage segmentation homogeneity and quality. However, the datasets composed mainly of healthy volunteers are disadvantageous to the generalization ability of the model in wrist disease diagnosis. The network needs to be optimized on datasets that contain more patients to suit real clinical conditions.

### 4.2 DL for wrist joint disease diagnosis

As the core operator of edge segmentation, the Canny operator has good anti-noise performance and accurate edge positioning function, but when applied to complex images, its edge detection performance will decline [66]. Due to the complex wrist structure, it is challenging to achieve automated segmentation because of the artifacts and uneven grayscale produced by MRI scanning [67]. Therefore, Li et al. [68] first used the Canny operator to extract the edge of MRI images from patients with wrist injuries, then classified the extracted edges by particle swarm optimization-support vector machine (PSO-SVM) algorithm, achieving a sound effect of edge detection, thus diagnosed wrist joint injury (WJI) with the diagnostic accuracy of 0.9413.

**Fig. 2:**
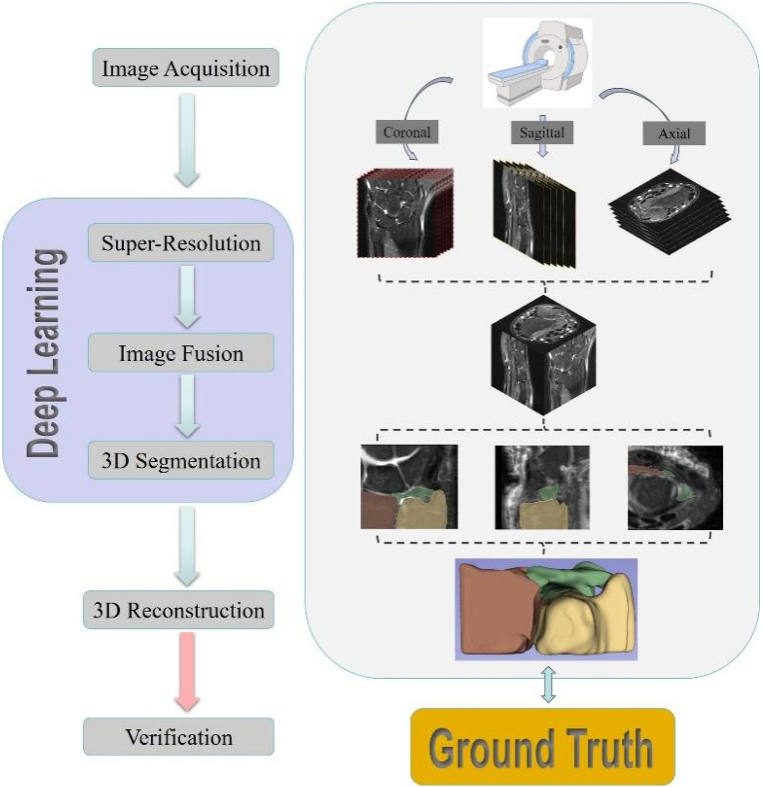
Process of TFCC 3D reconstruction. The DL image analysis method for the anatomical structure of the wrist in MRI realizes the intelligent processing of MRI images and the intelligent reconstruction of the TFCC.

Triangular fibrocartilage complex (TFCC) injury is a common type of WJI. The results of Palmer 1B TFCC injury prediction based on ultrasound combined with an AI model were exciting and promising [69]. Lin et al. [70] found that 3D MRNet outperformed 2D ResNet50 in the diagnostic accuracy (AUC of 0.871 vs 0.809) of TFCC injury, which is related to the fact that 3D MRNet architecture is based on an ensemble method combining models to solve a single prediction problem. Therefore, 3D CNN architecture can suitably process MRI scans comprising 3D data from three views (Fig. 2). Intercarpal ligament injury usually leads to carpal bone misalignment, the most common cause of wrist instability. Fast low-angle shot (FLASH) is a commonly used real-time MRI sequence for dynamic evaluation of wrist function. It has the advantage of nonlinear inverse reconstruction with temporal regularization, maintaining good spatial and temporal resolution at the same time [71]. Radke et al. [72] realized automatic wrist movement quantification by combining morphological FLASH sequences, standardized active radioulnar movement, and U-Net segmentation. The proposed method utilized the semantic segmentation approach based on CNN to realize the dynamic evaluation of wrist function on real-time MRI and quantify the carpal configuration (in terms of scapholunate and lunotriquetral gap widths). However, the tiny and complex structure of wrist ligaments causes difficulty in MRI identification, which may lead to a low diagnostic accuracy of WJI based on MRI alone. Therefore, constructing an AI large model based on present history, physical examination, and imaging examination will be the trend of wrist disease diagnosis.

Rheumatoid arthritis (RA) is a chronic systemic disease characterized by inflammatory synovitis. RA often affects the wrist and fingers with consequent joint deformity and loss of function. Typical MRI findings associated with RA include synovitis, marrow edema, erosions, and malalignment/subluxation, as seen on clinical assessment. Marrow edema and erosions on MRI can be evaluated by an automatic segmentation algorithm based on Watershed and Multi Otsu [73]. The atlas-based segmentation and marker-based watershed proposed by Aizenberg et al. [67,74] successfully segmented ten extensor and flexion tendons and marrow edema regions and quantified RA tenosynovitis and bone marrow edema of the wrist joint. Although the existence of blood vessels and synovitis causes some deviation from the quantitative results, these DL-based automatic quantization results could serve as future important biomarkers to assess the disease process of RA.

**Fig. 3:**
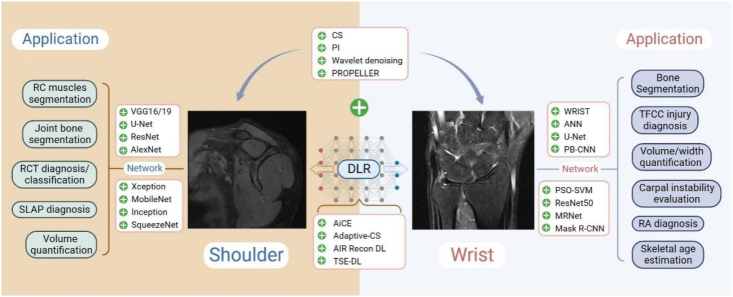
An overview of AI application in shoulder and wrist MRI. The middle lower part contains the deep learning image reconstruction (DLR) technique or algorithm, and the upper part contains the conventional image acceleration or denoising technique. The combination or independent application of the two techniques obtain a super-resolution MRI image and/or reduces the acquisition time. The side parts are the application of the DL network in MRI image segmentation or disease diagnosis.

In addition to diagnosing wrist diseases, DL-CNN has been used to evaluate pediatric bone age on hand X-rays. However, the study only compared the neural network model estimates regarding the standard bone ages and not the patient's chronological age [75]. Tang et al. [76] proved that ML can accurately estimate the chronological age by using independent bone maturity indexes based on artificial neural networks (ANN), such as height, weight, and bone marrow composition intensity quantified by MRI and TW3 bone age. Fig. 3 concludes the AI application in shoulder and wrist MRI.

## 5 Limitations and challenges of implementing AI in MRI

While AI is widely used in the field of medical imaging, there are several limitations of its application on MRI: 1) the engineer who writes the algorithm may add a personal understanding of the structure or disease into the algorithm, which may result in biases in structure identification and disease diagnosis due to insufficient understanding [77]; 2) some algorithms only suit for specific datasets, and the homogeneity will lead to overfitting of the training data, resulting in model offset; 3) the quality and heterogeneity of MRI images, the characteristics and complexity of anatomical structures, the scale and source of datasets, the definition of ground truth, and the interpretation of clinicians and radiologists will all limit the generalization ability of AI models [33,36,37,39,51,72,78]. This narrow, task-specific approach is limited by training datasets and pre-given tasks, and it cannot accomplish other tasks without retraining the model on another dataset. A new paradigm in medical AI known as generalist medical AI (GMAI) is emerging, which will be capable of performing a diverse set of tasks using minimal or no task-specific labeled data [79]. GMAI is constructed through self-supervision on large, diverse datasets, allowing for flexible interpretation of various medical data, such as image data, electronic medical records, and blood test results.

However, the application of GMAI models in the field of MRI imaging will face many challenges. Firstly, due to their unprecedented versatility, GMAI models will be difficult to validate. Secondly, GMAI models can handle exceptionally complex inputs and outputs, making it harder for clinicians and radiologists to ascertain their correctness. In such cases, the multidisciplinary panel will probably be employed. Thirdly, biases caused by the GMAI model will be more pronounced due to the larger scale and complexity of the training datasets [80,81]. Therefore, GMAI models must be thoroughly validated when employed in a specific task, such as RCT diagnostics. Fourthly, developing and using GMAI models poses severe risks to patient privacy. By employing deidentification and limiting the amount of information on MRI images, the damage caused by exposed private data can be reduced. Lastly and most importantly, the demand for unprecedented medical data poses a particular challenge to data collection for GMAI development. Multimodal self-supervision techniques can train models on datasets of multiple different medical modalities, thus reducing the need for large and expensive datasets [82]. Additionally, large-scale data-sharing efforts will be critical in implementing GMAI in MRI [83,84].

Since the advent of AI, ethical issues have been a focal point of societal concern. Ethical concerns of AI in medical application mainly revolve around general issues of AI application and unique issues related to medical treatment, of which the most universal is data privacy. Medical data from medical imaging usually contains patient identity information, health status, disease diagnosis and treatment, involving patient privacy and possessing exceptional sensitivity and important value [85]. In the context of big data, besides taking technical measures such as "anonymization" and encrypted storage, strengthening data management is the top priority to strike a balance between medical data sharing and privacy security. The security management of medical image data includes data collection, storage, mining, application, and transmission involving medical institutions, AI suppliers, medical information management departments, and other related units. Therefore, relevant departments should establish security management systems and operating procedures, strengthen supervision, build a credible network security environment, reasonably use medical image data following regulations, and strictly standardize data use rights and data access control to protect patient privacy and data security [86,87]. Apart from data privacy issues, challenges in the application of AI in medical imaging include the issues of medical security, responsibility definition, patient trust arising from AI misjudgments, unfair benefits from AI application biases or discrimination, and the future positioning of clinicians and radiologists. Therefore, close attention must be paid to the ethical issues and security risks caused by AI, and measures should be taken to make AI application in medical imaging always develop in a direction beneficial to human health.

## 6 Prospects and conclusions

Wide-bore 1.5-T and 3.0-T MRI systems have become mainstream for clinical musculoskeletal MRI. With the maturation application of 7.0-T MRI on the knee, images with higher SNR will help clinicians observe tiny structures and occult lesions, especially for wrist ligaments. They may enable novel quantitative MRI applications, such as nonproton imaging [88]. In addition, with the development of radiofrequency coils dedicated to musculoskeletal MRI, the optimization of synthetic MRI and MR fingerprinting approaches that can provide qualitative and quantitative pathological information will promote the progress of MRI imaging of upper extremity joints. Compared with the widely used and increasingly mature CNN architecture, Transformer is a newly developed DL model for image processing based on a self-attention mechanism in recent years, which is different from the convolution structure of CNN based on local connection and weight sharing [89]. Although the structure of the Transformer model is complex and requires many parameters and large-scale datasets, it can handle data noise and deformation better than the CNN structure. Previous studies have combined Transformer with CNN or GAN for knee joint MRI, and its segmentation performance and image quality are better than the most advanced CNN model [[90], [91], [92], [93], [94]]. However, only one study applied U-Net to segment supraspinatus tendon (ST) and Swin Transformer to determine whether ST tear presents in upper extremity joint MRI [57]. In future research, attention should be paid to reducing the computational complexity and training load and enhancing the accuracy and interpretability of the DL algorithm in diagnosing musculoskeletal diseases based on MRI. The continuous optimization of the Transformer will bring radical changes to the upper extremity joint MRI imaging technology and lesion detection. Additionally, we can not ignore the challenges and opportunities brought by GMAI in MRI imaging technology and musculoskeletal disease diagnosis.

This article has provided a comprehensive overview of AI's current research and application in MRI examinations of the upper extremities. The benefits of DL-based accelerated MRI and super-resolution imaging techniques can reduce MRI scan duration and patient discomfort while improving image quality and diagnosis. This would also allow better allocation of healthcare resources to help facilitate patient throughput and reduce the workload on healthcare professionals. While the article discussion revolves around the application of AI to upper extremity MRI, the authors expect that AI will expand its reach to a wider variety of disease processes in the future. As joint diseases are complex and diverse, further research would be needed to apply AI to a broader disease spectrum.

## Ethics statement

Informed consent was not required for this study because it is a review and no subjects were investigated.

## Data availability statement

No data was used for the research described in the article.

## CRediT authorship contribution statement

**Wei Chen:** Writing – original draft, Investigation, Conceptualization. **Lincoln Jian Rong Lim:** Writing – review & editing. **Rebecca Qian Ru Lim:** Resources. **Zhe Yi:** Writing – review & editing, Investigation. **Jiaxing Huang:** Writing – review & editing. **Jia He:** Writing – review & editing. **Ge Yang:** Supervision, Resources. **Bo Liu:** Supervision, Resources, Project administration, Funding acquisition, Conceptualization.

## Declaration of competing interest

The authors declare that they have no known competing financial interests or personal relationships that could have appeared to influence the work reported in this paper.
